# Hsa_circ_0001944 Regulates FXR/TLR4 Pathway and Ferroptosis to Alleviate Nickel Oxide Nanoparticles-Induced Collagen Formation in LX-2 Cells

**DOI:** 10.3390/toxics13040265

**Published:** 2025-03-31

**Authors:** Haodong Zhou, Qingyang Chen, Lijiao Ma, Gege Li, Xi Kang, Jiarong Tang, Hui Wang, Sheng Li, Yingbiao Sun, Xuhong Chang

**Affiliations:** 1 School of Public Health, Lanzhou University, Lanzhou 730000, China; 220220913241@lzu.edu.cn (H.Z.); 220220912231@lzu.edu.cn (Q.C.); mlijiao2023@lzu.edu.cn (L.M.); liggcharon@163.com (G.L.); kangx2023@lzu.edu.cn (X.K.); tangjr01@126.com (J.T.); huiwang@lzu.edu.cn (H.W.); sunyb@lzu.edu.cn (Y.S.); 2 The No. 2 People’s Hospital of Lanzhou, Lanzhou 730046, China; lisheng76@sohu.com

**Keywords:** NiONPs, collagen deposition, ferroptosis, FXR/TLR4, hsa_circ_0001944

## Abstract

Nickel oxide nanoparticles (NiONPs) can induce liver fibrosis, and their mechanism may be related to non-coding RNA, nuclear receptor signal transduction and ferroptosis, but the regulatory relationship between them is not clear. In this study, we aimed to investigate the role of hsa_circ_0001944 in regulating the Farnesol X receptor (FXR)/Toll-like receptor 4 (TLR4) pathway and ferroptosis in NiONPs-induced collagen deposition. We observed decreased FXR expression, increased TLR4 expression and alterations in ferroptosis features in both the rat liver fibrosis and the LX-2 cell collagen deposition model. To investigate the regulatory relationship among FXR, TLR4 and ferroptosis, we treated LX-2 cells with FXR agonist (GW4064), TLR4 inhibitor (TAK-242) and ferroptosis agonist (Erastin) combined with NiONPs. The results showed that TAK-242 alleviated collagen deposition by increasing ferroptosis features. Furthermore, GW4064 reduced the expression of TLR4, increased the ferroptosis features and alleviated collagen deposition. The results indicated that FXR inhibited the expression of TLR4 and enhanced the ferroptosis features, which were involved in the process of collagen deposition in LX-2 cells induced by NiONPs. Subsequently, we predicted that hsa_circ_0001944 might regulate FXR through bioinformatics analysis, and found NiONPs reduced the expression of hsa_circ_0001944 in LX-2 cells. Overexpression of hsa_circ_0001944 increased FXR level, reduced TLR4 level, increased the ferroptosis features and alleviated collagen deposition in LX-2 cells. In summary, we demonstrated that hsa_circ_0001944 regulates the FXR/TLR4 pathway and ferroptosis alleviate collagen formation induced by NiONPs.

## 1. Introduction

In recent years, nickel oxide nanoparticles (NiONPs) have been widely used in industrial fields due to their unique properties [1,2]. NiONPs can enter the body through the respiratory system, digestive tract and other pathways, deposit in the liver and cause liver damage [3]. Studies have shown that NiONPs can induce liver fibrosis by inducing inflammation in liver cells [4]. Our previous research found that NiONPs can increase the expression of pro-inflammatory cytokines and collagen fibers around the portal vein bundles in the liver tissue of male Wistar rats, as well as the protein expression levels of type I collagen (Col-I) and type III collagen (Col-III) [5,6]. Hepatic stellate cells (HSCs) play an important role in the process of liver fibrosis. When liver injury occurs, HSCs are activated and transformed into myofibroblasts, secreting a large amount of extracellular matrix (ECM), which leads to liver fibrosis [7,8]. Our previous research found that NiONPs can increase the expression of collagen type I alpha 1 chain (COL1A1) in human HSCs (LX-2) [9], but the specific mechanism is still unclear. Recent studies have shown that the activation of HSCs may be related to biological processes such as non-coding RNA, nuclear receptor signal transduction, and ferroptosis [10,11,12], but the regulatory relationship between them is not clear This study will focus on FXR, TLR4 and ferroptosis as the starting points to explore their mutual regulatory effects in the process of LX-2 cell activation and collagen deposition induced by NiONPs.

Ferroptosis was an iron-dependent cell death characterized by lipid peroxidation, and its role in liver diseases has attracted attention in recent years [13]. Ferroptosis plays an important role in the collagen deposition of HSCs [14]. Studies have shown that activation of ferroptosis in mouse HSCs using Erastin increased reactive oxygen species (ROS) and malondialdehyde (MDA) levels, decreased glutathione peroxidase 4 (GPX4), glutathione (GSH) and COL1A1 levels and alleviated liver fibrosis [15]. Wogonoside activated ferroptosis by decreasing the levels of solute carrier family 7 member 11 (SLC7A11), GPX4, and GSH in mouse HSC-T6 cells, while reducing the levels of α-smooth muscle actin (α-SMA) with COL1A1 and alleviating liver fibrosis [16]. Our previous study showed that NiONPs caused liver fibrosis with changes in ferroptosis-related markers (glutathione peroxidase (GPx), superoxide dismutase (SOD) and total antioxidant capacity (T-AOC)) in rats [17]. Whether ferroptosis is involved in the process of NiONPs-induced collagen deposition in LX-2 cells needs to be further studied.

Toll-like receptor 4 (TLR4), as a key member of the toll-like receptor (TLR) family, plays an important role in regulating hepatocyte inflammation and collagen deposition [18,19]. Lipopolysaccharide (LPS) induced hepatocyte collagen deposition in mice by increasing the TLR4 expression to increase the levels of interleukin-1β (IL-1β) and tumor necrosis factor alpha (TNF-α) [20]. CCl4 induced collagen deposition by increasing the expression of TLR4 to activate NF-κB signaling in LX-2 cells [21]. TLR4 caused collagen deposition by regulating ferroptosis in hepatocytes [22,23]. High glucose induced collagen deposition by increasing TLR4 expression to activate ferroptosis in human normal liver cells (LO2) [24]. However, the relevant mechanism in hepatic stellate cells is still unclear. Our previous study found that NiONPs caused liver fibrosis by activating NF-κB signaling in rats [5], and caused collagen deposition by activating the Jun amino-terminal kinase (JNK) pathway in LX-2 cells [9]. TLR4 was an upstream molecule regulating the NF-κB and JNK signaling pathways, and its roles was unclear in the process of NiONPs-induced collagen deposition.

Farnesol X receptor (FXR), an important functional nuclear receptor, participates in the process of collagen deposition by regulating the activity of HSCs [12]. LPS induced collagen deposition by reducing the level of FXR to activate mouse HSCs and increase the COL1A1 content [25]. A choline-deficient high-fat diet promoted collagen deposition by reducing the expression of FXR and its ligand (small heterodimer partner (SHP)) to activate rat HSCs and increase the levels of hydroxyproline (Hyp) and COL1A1 [26]. FXR participated in liver cell injury by regulating TLR4 signaling and ferroptosis. Hypoxia-reoxygenation treatment caused hepatocytes injury in mice by reducing the level of FXR to activate TLR4 signaling and induce inflammation (activated NF-κB signaling and increased IL-1β expression) [27]. A methionine/choline-deficient diet induced liver fibrosis by reducing FXR and its ligand SHP levels to activate ferroptosis of hepatocytes in mice [28]. However, the relevant mechanism in hepatic stellate cells is still unclear. Our previous study found that NiONPs caused disturbances in bile acid metabolism (the serum cholic acid (CA) and deoxycholic acid (DCA) and liver cholesterol 7α-hydroxylase (CYP7A1) expression were decreased) in rats [29]. As an important molecule regulating bile acid metabolism, whether FXR was involved in the process of NiONPs-induced collagen deposition in LX-2 cells needs further study.

Circular RNAs (circRNAs) were a kind of single-stranded circular non-coding RNA produced by back-splicing [30], regulated in the occurrence and development of collagen deposition in hepatocytes by acting as a sponge for microRNAs (miRNAs) to increase the level of target genes [31,32]. LPS induced collagen deposition by increasing the expression of CircPWWP2A, which promoted the activation of LX-2 cells and increased the expression of COL1A1 and α-SMA by acting as a sponge for miR-203 and miR-223 to upregulate follistatin-like protein 1 (FSTL1) and TLR4 levels, respectively [33]. Overexpression of circCREBBP suppressed carbon tetrachloride (CCl4)-induced collagen deposition by acting as a sponge for hsa-miR-1291 to increase Left-Right Determination Factor 2 (LEFTY2) expression to inhibit LX-2 cells activation and decrease COL1A1 level [34]. Our previous study showed that NiONPs caused differential expression of circRNAs in liver fibrosis rats [9]. The mechanism of circRNAs needs further study in the process of NiONPs-induced collagen deposition.

In this study, we hypothesized that NiONPs caused collagen deposition in LX-2 cells by inhibiting the FXR/TLR4 signaling pathway and attenuating the ferroptosis signature. Therefore, LX-2 cells were treated with NiONPs, GW4064 (FXR agonist), TAK-242 (TLR4 inhibitor) and Erastin (ferroptosis agonist) to investigate the effect of the FXR/TLR4 signaling pathway and ferroptosis on collagen deposition. At the same time, bioinformatics methods were used to predict circRNA (circ_0001944) that may target FXR, and their regulatory relationship with collagen deposition in LX-2 cells was verified.

## 2. Material and Methods

### 2.1. Characterization of NiONPs and Sample Preparation

NiONPs were obtained from ST-nano science and technology Co., Ltd. (Shanghai, China); the average particle size was 20 nm, hydrodynamic size was 244.5 nm. The results of the endotoxin examination were negative. The specific methods for NiONPs characterization and endotoxin detection were described in our previous studies [5]. After high pressure steam sterilization, NiONPs were dissolved in 9% normal saline or DMEM and sonicated by the ultrasonic homogenizer (Cole-Parmer, CP750, Vernon Hills, IL, USA) before treatment to prevent aggregation. The prepared NiONPs suspension was diluted to the experimental concentration using 9% normal saline or DMEM, which was used to treat rats and LX-2 cells, respectively.

### 2.2. Construction of Animal Models

In this study, the rat model of liver fibrosis induced by NiONPs was established based on the previous research conducted by our group. The specific methods are as follows. Forty adult male Wistar rats (190–230 g) of grade specific pathogen-free (SPF) were obtained from Experimental Animal Center of Lanzhou University (SCXK2018–0002). The study was conducted according to the guidelines of the Declaration of Helsinki, and approved by the Institutional Review Board of School of Public Health, Lanzhou University (LRB18120201). We performed animal experimental procedures in strict accordance with the guidelines approved by the Experimental Animal Ethics Committee of Lanzhou University to ensure proper animal care and welfare. All rats were housed in SPF animal cages and fed standard pellet food and drinking water. After one week of adaptive feeding, the rat liver fibrosis model was established by the routine method in our laboratory [6]. After the model was successfully constructed, the rat liver was stored in liquid nitrogen for subsequent transcriptome sequencing and other experiments.

### 2.3. Transcriptome Sequencing of Liver Tissue

Transcriptome sequencing was used to detect the changes of gene expression in the liver tissue of rats after NiONPs treatment. The liver tissue was sent to Hangzhou Lianchuan Biological Co., Ltd. for transcriptome sequencing. The Kyoto Encyclopedia of Genes and Genomes (KEGG) pathway database and Gene Ontology (GO) database were used for enrichment and pathway analysis. *p* < 0.10 was considered statistically significant.

### 2.4. Bioinformatic Prediction

The miRNAs targeting FXR were predicted by the miRDB website (https://mirdb.org/mining.html, accessed on 1 January 2024) and mirDIP website (https://ophid.utoronto.ca/mirDIP/index.jsp#r, accessed on 1 January 2024). The Circular RNA interactome website (https://circinteractome.nia.nih.gov/index.html, accessed on 1 January 2024) was used to predict the circRNAs targeting miR-1225-5p.

### 2.5. Cell Culture

LX-2 cells were purchased from Pratzer Biological Co, Ltd., (Changsha, China), and DMEM (Gibco, Thermo Fisher Scientific, NY, USA) with 10% FBS (Viva Cell Biosciences, Shanghai, China) and 1% antibiotic/antimycotic solution (Viva Cell Biosciences, Shanghai, China) was used to culture the cells. The cell culture incubator was kept at 37 °C temperature with 5% CO_2_ and 95% humidity.

### 2.6. Treatment of LX-2 Cells

#### 2.6.1. Establishment of Collagen Deposition Model

The method in Section 2.1 was used to configure the NiONPs suspension for treatment of the LX-2 cells. According to the previous study [9], the LX-2 cells were treated with 0, 1.25, 2.5 and 5 μg/mL NiONPs suspension for 12 h to establish the excessive deposition model of cell collagen.

#### 2.6.2. Drug Treatment

To further investigate the role of the FXR/TLR4 pathway and ferroptosis in NiONPs-induced collagen deposition, LX-2 cells were treated with 5 μg/mL NiONPs combined with GW4064 (FXR agonist, 5 μM, Cat No. B1527, APExBIO Technology, Houston, TX, USA), TAK-242 (TLR4 inhibitor, 10 μM Cat No. A3850, APExBIO Technology, Houston, TX, USA) and Erastin (Ferroptosis agonists, 10 μM, Cat No. GC16630, GLPBIO, Montclair, CA, USA) for 12 h.

#### 2.6.3. Overexpression of circ_0001944

The circ_0001944 overexpression plasmid was constructed, and the gene sequence was obtained from NCBI GenBank (Position: chrX: 130883333-130928494). The length of the gene was 1096 bp, the cloning vector was GV727, and the cloning sites were AgeI and BamHI (Figure S1A,B; Table S1). LX-2 cells were seeded in 6-well plates and the circ_0001944 overexpression plasmid (2.5 μg/well) or empty plasmid (2.5 μg/well) was mixed with M5 HiPer Lipo2000 transfection reagent (7.5 μL/well) (MF135-01, Mei5bio, Beijing, China) and transfected into the cells for 12 h. After decanting the transfection reagent, the cells were allowed to recover for 24 h by adding medium containing serum and were treated with NiONPs (5 μg/mL) for 12 h.

### 2.7. Cell Migration Assay

The LX-2 cells were seeded into a 12-well plate, and a 10 μL gun tip was used to draw a vertical line in the center of each well. The old medium was discarded and cell debris was removed by washing with PBS three times. Pictures were taken under a microscope at 0, 12, 24 and 36 h after addition of the configured reagents. Image J was used to analyze the pictures and calculate the scratch healing rate.

### 2.8. Cell Cycle Detection

The LX-2 cells were seeded with T25 cell culture flasks and were treated using the instructions of the cell cycle assay kit (E-CK-A352, Elabscience, Wuhan, China). The cell cycle changes of the different treatment groups were detected by flow cytometry (BD Accuri^®^ C6 Plus, BD, NJ, USA).

### 2.9. Real-Time Fluorescent Quantitative PCR (RT-qPCR)

Total RNA was extracted from LX-2 cells using RNAiso Plus (TaKaRa, Tokyo, Japan). The extracted RNA was reverse transcribed into cDNA using PrimeScript™ RT reagent Kit with gDNA Eraser (TaKaRa, Tokyo, Japan). Fluorescence quantification of the target genes was performed using STBR^®^ Premix Ex Taq II (Tli RNaseH Plus) (TaKaRa, Tokyo, Japan) reagent. The reaction conditions were as follows: 40 cycles of predenaturation 95 °C, 30 s → denaturation 95 °C, 5 s → annealing 55 °C ~65 °C, 30 s → extension 72 °C, 30 s. The primer information is included in Table 1. GAPDH was used as the reference gene for circRNA and U6 as the reference gene for microRNA. Gene expression levels were analyzed using the 2^−△△t^ method.

### 2.10. Western Blotting

Liver tissue and LX-2 cells were lysed using RIPA lysis buffer (Boster Bio, Pleasanton, CA, USA) in combination with protease inhibitors (Boster Bio, CA, USA) and phosphatase inhibitors (APExBIO, Houston, TX, USA) to release proteins. Supernatants were collected by centrifugation and protein quantification was measured by Pierce BCA Protein Assay Kit (#23227, Thermo Scientific, Bohemia, NY, USA). Quantitative protein samples were mixed with 5 × SDS loading buffer (Boster Bio, CA, USA), boiled in boiling water for 10 min, and stored at −80 °C. Total proteins were separated on SDS-PAGE gels and transferred to polyvinylidene difluoride (PVDF) membranes (Millipore, Burlington, MA, USA). After blocking in 5% nonfat milk in TBST solution for 2 h, membranes were incubated with primary antibodies overnight at 4 °C. The membranes bound to the primary antibody were washed with TBST and incubated with the secondary antibody for another 1.5 h at room temperature before being washed again. Antibody information is shown in Table 2. The target protein bands were visualized in the molecular imaging instrument ChemiDoc XRS system (Bio-Rad, Hercules, CA, USA) using the super electrochemiluminescence (ECL) and kit (UElandy, Beijing, China).

### 2.11. Fluorescent Probe

The fluorescence probe method is mainly used to detect the content of reactive oxygen species (ROS) and glutathione (GSH) in cells. LX-2 cells were seeded in six-well plates and treated with 0, 1.25, 2.5 and 5 μg/mL NiONPs for 12 h according to the Reactive Oxygen Species detection kit (Beyotime, Shanghai, China). The old cell medium was removed and 1 mL of diluted DCFH-DA (10 μM) was added. The cells were incubated in a cell incubator at 37 °C for 20 min and then washed three times with PBS buffer. Then, the treated cells were observed and photographed under a fluorescence microscope.

In this study, a novel probe (BTFMD, Lanzhou University, Lanzhou, China) was used to detect intracellular GSH content [35,36]. The probe can be recognized by three biothiols, GSH, Cys and Hcy, and release the green fluorophore BTFM-OH, while Cys and Hcy further cyclize with the aldehyde group of the fluorophore and then turn off the green fluorescence to achieve selective detection of GSH. The pretreatment of cells was the same as above, the BTFMD probe was diluted to a working concentration of 10 μM and added to the cells for 1 h incubation. Finally, the treated cells were washed with PBS buffer and observed under a fluorescence microscope and photographed.

### 2.12. Determination of Intracellular Iron Ions

The contents of total iron ion and ferrous ion were determined according to the instructions of the total cell iron colorimetry detection kit (E-BC-K880-M, Elabscience, Wuhan, China) and the cell ferrous ion colorimetry detection kit (E-BC-K881-M, Elabscience, Wuhan, China), respectively. Then, the proportion of ferrous ion was calculated in the total iron ion. The detection range of the two kits was 0.4–50 μM/L, and the sensitivity was 0.4 μM/L.

### 2.13. Statistical Analysis

SPSS 27.0 Statistics version (IBM, Armonk, NY, USA) was used for statistical analysis. Data are presented as mean ± standard deviation (SD). One-way analysis of variance (ANOVA) and Fisher’s least significant difference (LSD) method were used for post hoc tests. *p* < 0.05 results were considered statistically significant.

## 3. Results

### 3.1. NiONPs Affected the FXR/TLR4 Pathway, Ferroptosis and Inflammation in Rat Liver Tissue

**Figure 1:**
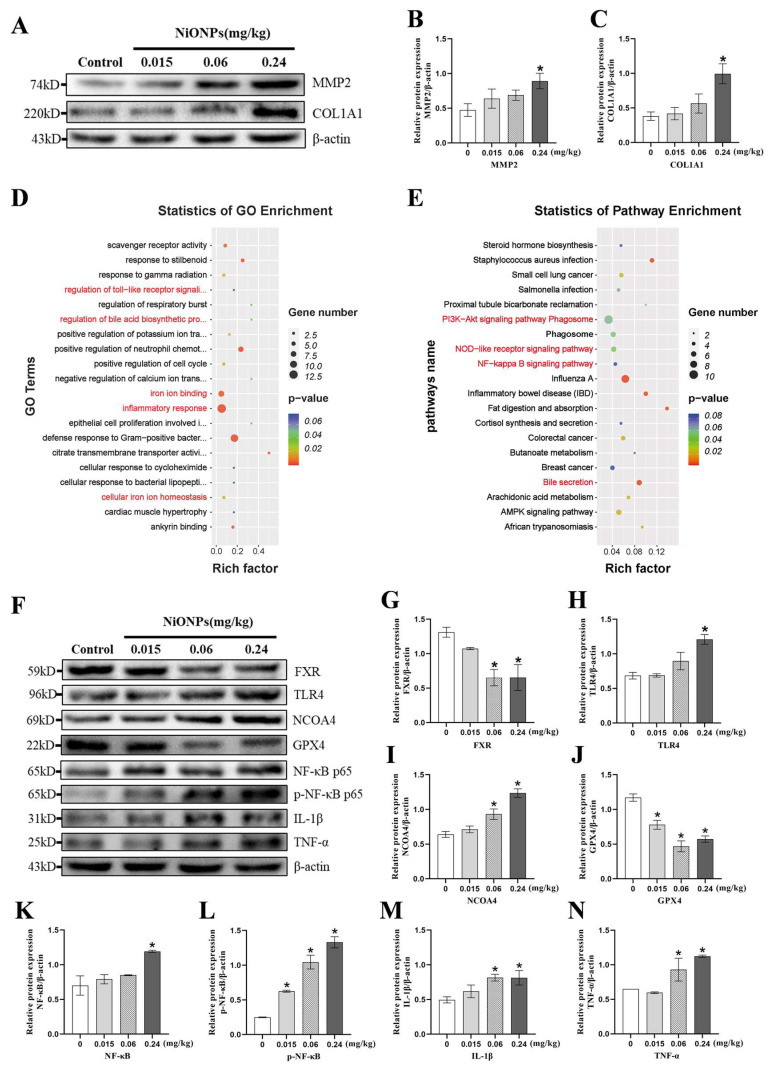
Nickel oxide nanoparticles (NiONPs) affected the transcriptome, Farnesol X receptor (FXR)/Toll-like receptor 4 (TLR4) pathway, ferroptosis and inflammation in rat liver tissue. (**A**–**C**) The protein expression levels of MMP2 and COL1A1. (**D**) GO functional enrichment analysis. (**E**) KEGG pathway enrichment analysis. (**F**–**N**) The protein expression levels of the FXR/TLR4 pathway, inflammation and ferroptosis indicators. * *p* < 0.05, compared to the control group.

NiONPs increased the protein contents of MMP2 and COL1A1 (Figure 1A–C) in liver tissue. GO analysis showed that NiONPs caused changes in biological functions such as Toll-like receptor signaling pathway regulation, bile acid metabolism, cellular iron homeostasis and inflammatory response (Figure 1D and Figure S2). KEGG analysis showed that NiONPs activated PI3K-AKT, NOD-like, NF-κB and bile secretion signaling pathways (Figure 1E). Therefore, the related biological functions (ferroptosis, inflammation) and the key molecules of signaling pathways (FXR and TLR4) were selected for subsequent studies. In addition, 0.24 mg/kg NiONPs increased the expression levels of TLR4, NCOA4, NF-κB, p-NF-κB, IL-1β and TNF-α, and decreased the expression levels of FXR and GPX4 (Figure 1F–N). The results suggested that NiONPs affected the FXR/TLR4 pathway, inflammation and ferroptosis in rat liver tissue.

### 3.2. NiONPs Caused Collagen Deposition and the Changes in FXR/TLR4 Signaling Pathway, Ferroptosis and Inflammation in LX-2 Cells

**Figure 2:**
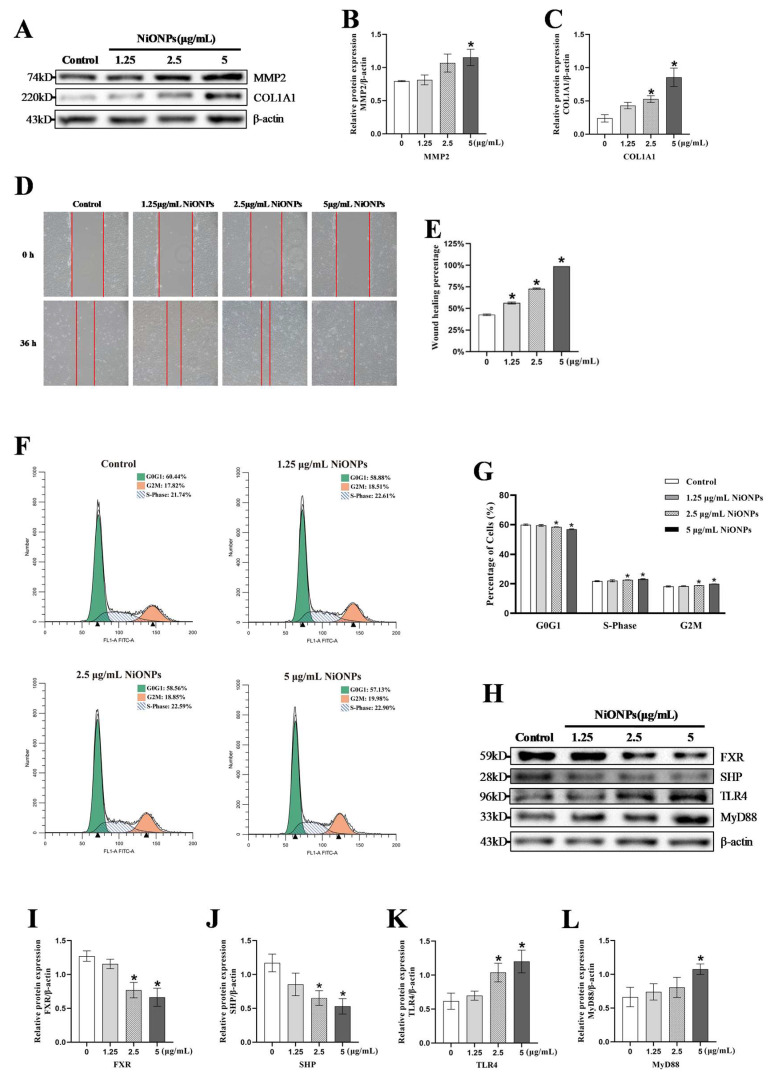
Nickel oxide nanoparticles (NiONPs) caused excessive collagen deposition and changes in Farnesol X receptor (FXR)/Toll-like receptor 4 (TLR4) signaling pathway in LX-2 cells. (**A**–**C**) Protein expression levels of MMP2 and COL1A1. (**D**,**E**) The scratch healing test (×100, n = 3). (**F**,**G**) Cell cycle assay. (**H**–**L**) Protein expression levels of FXR, SHP, TLR4 and MyD88. * *p* < 0.05, compared to the control group.

The 5 μg/mL NiONPs increased the levels of COL1A1 and MMP2 and caused collagen deposition in LX-2 cells (Figure 2A–C). The scratch healing rate of LX-2 cells (Figure 2D,E and Figure S3A) and the proportion of S and G2 phases were increased (Figure 2F,G), indicating that NiONPs enhanced the proliferation and migration ability of LX-2 cells. Compared to the control group, NiONPs decreased FXR and SHP protein contents, and increased TLR4 and MyD88 protein contents (Figure 2H–L). Those results indicated that NiONPs caused the excessive collagen deposition and activated the FXR/TLR4 signaling pathway.

**Figure 3:**
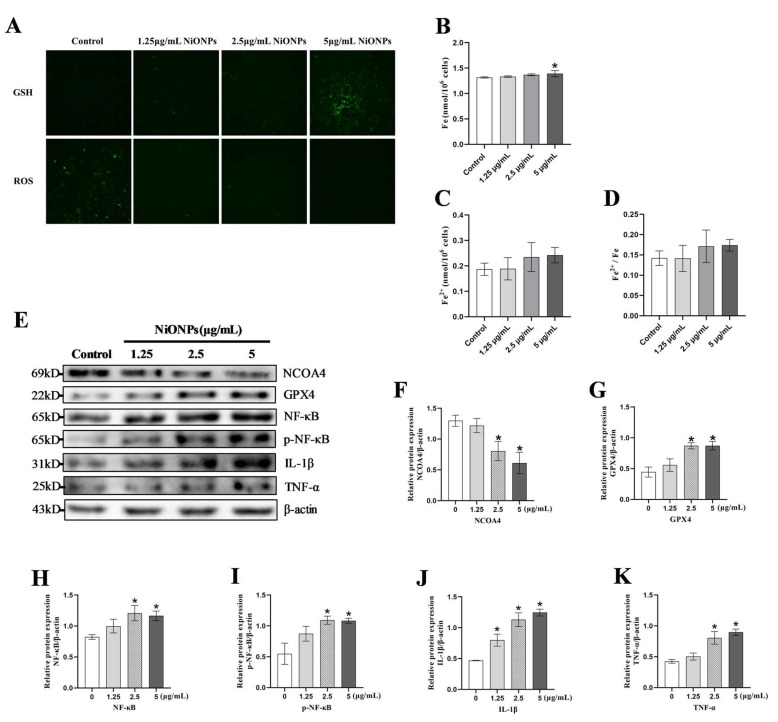
Nickel oxide nanoparticles (NiONPs) caused ferroptosis and inflammation-related indicators in LX-2 cells. (**A**) Cell content of GSH and ROS fluorescent probe test (×200, n = 3). (**B**–**D**) Intracellular iron ion content detection. (**E**–**K**) Protein expression levels of NCOA4, GPX4, NF-κB, p-NF-κB, IL-1β and TNF-α. * *p* < 0.05, compared to the control group.

Fluorescent probe results showed that LX-2 cells treated with 5 μg/mL NiONPs had increasing GSH levels and decreasing ROS levels (Figure 3A). Compared to the control group, NiONPs increased total intracellular iron levels, but neither the absolute nor the relative levels of ferrous ions were changed (Figure 3B–D). The protein contents of GPX4, NF-κB, p-NF-κB, IL-1β and TNF-α increased, while the protein contents of NCOA4 decreased at doses of 5 μg/mL NiONPs treatment (Figure 3E–K). Those results indicated that NiONPs caused the inflammation response, and attenuated the ferroptosis signature in LX-2 cells.

### 3.3. Ferroptosis Alleviated the Excessive Deposition of Collagen in LX-2 Cells Treated with NiONPs

**Figure 4:**
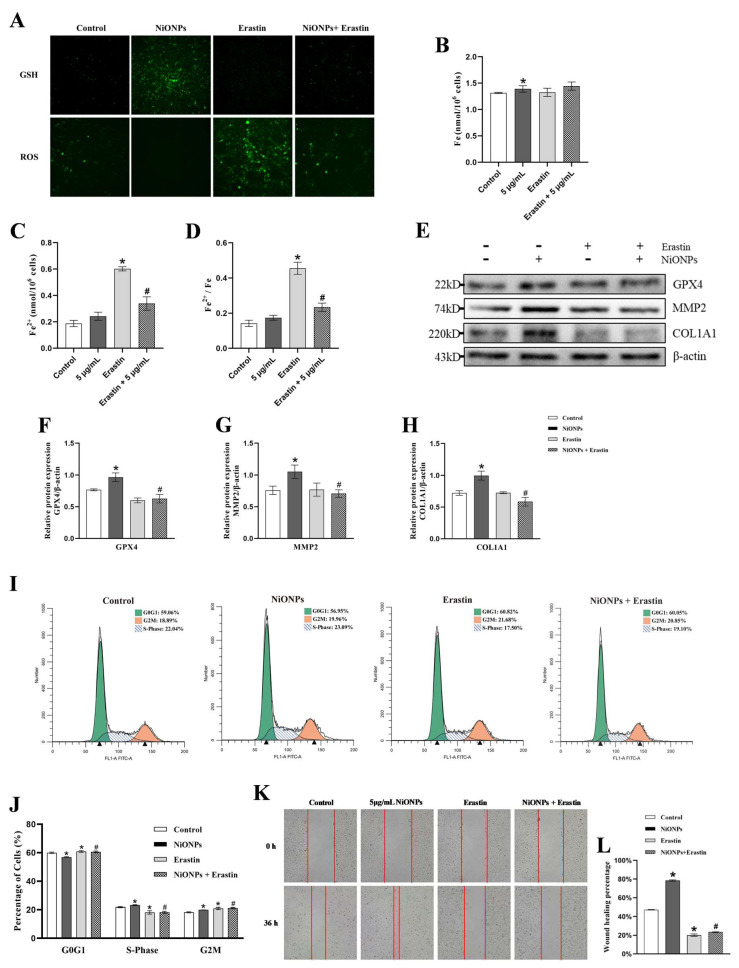
Activation of ferroptosis alleviated the excessive deposition of collagen in LX-2 cells treated with NiONPs. (**A**) GSH and ROS fluorescent probe test (×200, n = 3). (**B**–**D**) Intracellular iron ion content detection. (**E**–**H**) Protein expression levels of GPX4, MMP2 and COL1A1. (**I**,**J**) Cell cycle assay. (**K**,**L**) Scratch healing test (×100, n = 3). * *p* < 0.05, compared to the control group; ^#^ *p* < 0.05, compared with the 5 μg/mL NiONPs group.

Erastin inhibited the GSH upregulation and ROS downregulation, increased the intracellular iron ion content and the proportion of ferrous ion (Figure 4A–D) and decreased the expression level of GPX4 protein (Figure 4E,F). These results indicated that Erastin activated ferroptosis in LX-2 cells after NiONPs treatment. Compared with 5 μg/mL NiONPs group, the scratch healing rate (Figure 4K,L) and the proportion of S and G2 phases (Figure 4I,J), and the protein content of MMP2 and COL1A1 (Figure 4E,G,H) decreased in LX-2 cells treated with Erastin. Those results indicated that activation of ferroptosis alleviated NiONPs-induced collagen deposition in LX-2 cells.

### 3.4. Inhibition of TLR4 Signaling Pathway Alleviated the NiONPs-Induced Collagen Deposition and Increased the Ferroptosis Features

**Figure 5:**
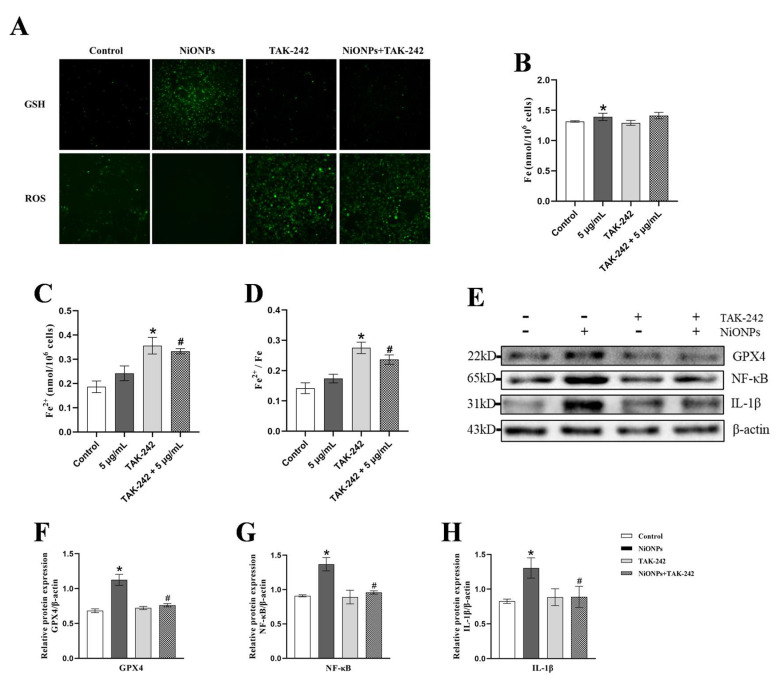
Inhibition of Toll-like receptor 4 (TLR4) signaling pathway increased the ferroptosis features in LX-2 cells. (**A**) GSH and ROS fluorescent probe test (×200, n = 3). (**B**–**D**) Intracellular iron ion content detection. (**E**–**H**) Protein expression levels of GPX4, NF-κB and IL-1β. * *p* < 0.05, compared to the control group; ^#^ *p* < 0.05, compared with the 5 μg/mL NiONPs group.

TAK-242 inhibited the GSH upregulation and ROS downregulation, and increased the intracellular iron ion content and the proportion of ferrous ion (Figure 5A–D). Compared with 5 μg/mL NiONPs group, the protein content of GPX4, NF-κB and IL-1β decreased in LX-2 cells treated with TAK-242 (Figure 5E–H). These results indicated that inhibition of TLR4 increased the ferroptosis features and alleviated the inflammatory response in LX-2 cells with NiONPs treatment.

**Figure 6:**
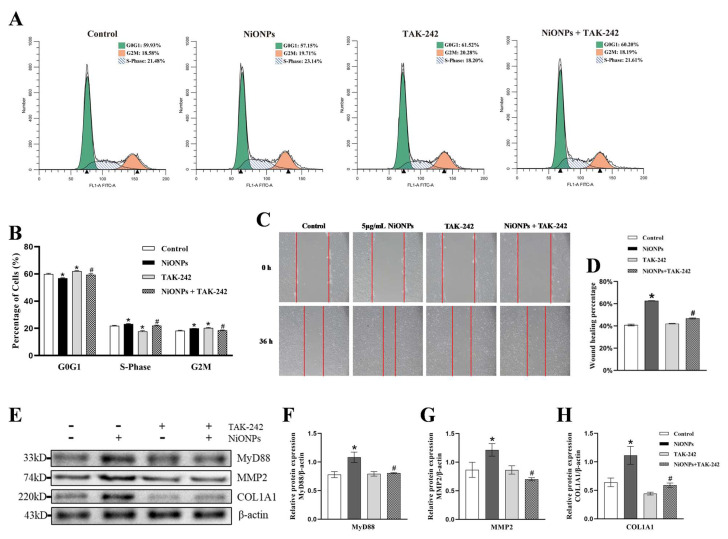
Inhibition of Toll-like receptor 4 (TLR4) signaling pathway alleviated the nickel oxide nanoparticles (NiONPs)-induced collagen in LX-2 cells. (**A**,**B**) Cell cycle assay. (**C**,**D**) Scratch healing test (×100, n = 3). (**E**–**H**) Protein expression levels of MyD88, MMP2 and COL1A1. * *p* < 0.05, compared to the control group; ^#^ *p* < 0.05, compared with the 5 μg/mL NiONPs group.

Cell scratch and cycle assay showed that TAK-242 reversed the increase in cell migration rate and the proportion of S and G2 phases (Figure 6A–D and Figure S3B). Compared with the 5 μg/mL NiONPs group, the protein content of MyD88, MMP2 and COL1A1 decreased in LX-2 cells treated with TAK-242 (Figure 6E–H). Those results indicated that inhibition of TLR4 alleviated the NiONPs-induced collagen deposition in LX-2 cells through increased ferroptosis and attenuated inflammation.

### 3.5. Activation of FXR Signaling Alleviated the NiONPs-Induced Collagen Deposition Through Inhibited TLR4 and Increased the Ferroptosis Features

**Figure 7:**
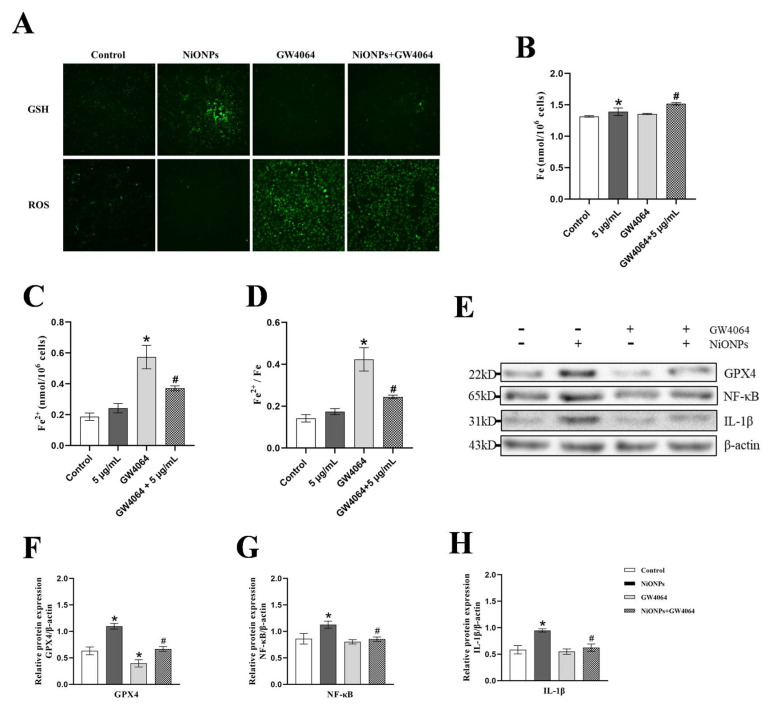
Activation of Farnesol X receptor (FXR) signaling pathway increased the ferroptosis features in LX-2 cells. (**A**) GSH and ROS fluorescent probe test (×200, n = 3). (**B**–**D**) Intracellular iron ion content detection. (**E**–**H**) Protein expression levels of GPX4, NF-κB and IL-1β. * *p* < 0.05, compared to the control group; ^#^ *p* < 0.05, compared with the 5 μg/mL NiONPs group.

GW4064 inhibited the GSH upregulation and ROS downregulation, and increased the intracellular iron ion content and the proportion of ferrous ion (Figure 7A–D). Compared with the 5 μg/mL NiONPs group, the protein content of GPX4, NF-κB and IL-1β decreased in LX-2 cells treated with GW4064 (Figure 7E–H). These results indicated that FXR increased the ferroptosis features and alleviated the inflammatory response in LX-2 cells with the NiONPs treatment.

**Figure 8:**
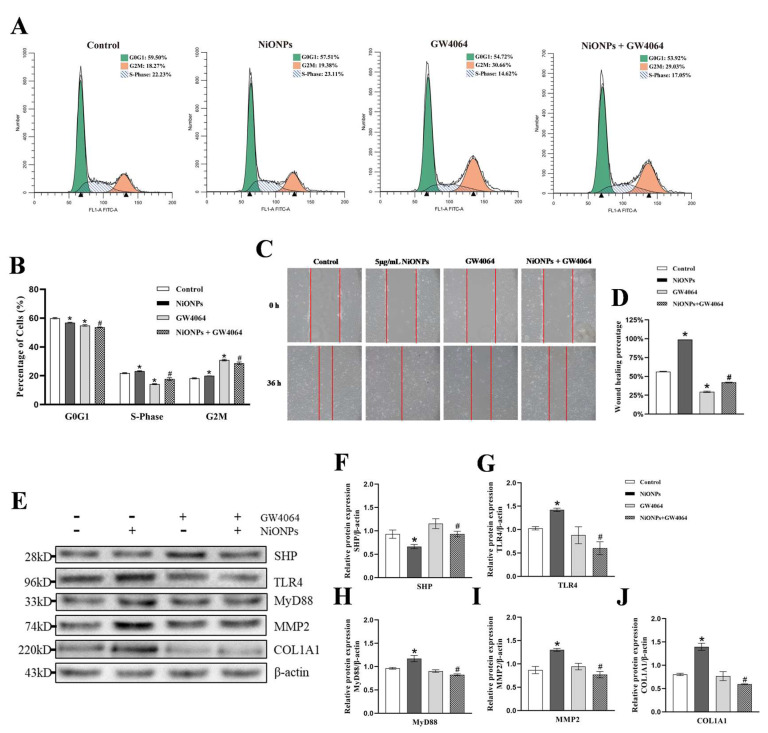
Activation of Farnesol X receptor (FXR) signaling pathway alleviated the nickel oxide nanoparticles (NiONPs)-induced collagen in LX-2 cells through inhibited Toll-like receptor 4 (TLR4). (**A**,**B**) Cell cycle assay. (**C**,**D**) Scratch healing test (×100, n = 3). (**E**–**J**) Protein expression levels of SHP, TLR4, MyD88, MMP2 and COL1A1. * *p* < 0.05, compared to the control group; ^#^ *p* < 0.05, compared with the 5 μg/mL NiONPs group.

Cell scratch and cycle assay showed that GW4064 reversed the increase in cell migration rate and the proportion of S and G2 phases (Figure 8A–D and Figure S3C). Compared with the 5 μg/mL NiONPs group, the protein content of SHP, TLR4, MyD88, MMP2 and COL1A1 decreased in LX-2 cells treated with GW4064 (Figure 8E–J). Those results indicated that activation of FXR alleviated the NiONPs-induced collagen deposition through inhibited TLR4, increased the ferroptosis features and attenuated inflammation in LX-2 cells.

### 3.6. Overexpression of hsa_circ_0001944 Alleviated the NiONPs-Induced Collagen Deposition Through Regulated FXR/TLR4 Signaling Pathway and Ferroptosis

**Figure 9:**
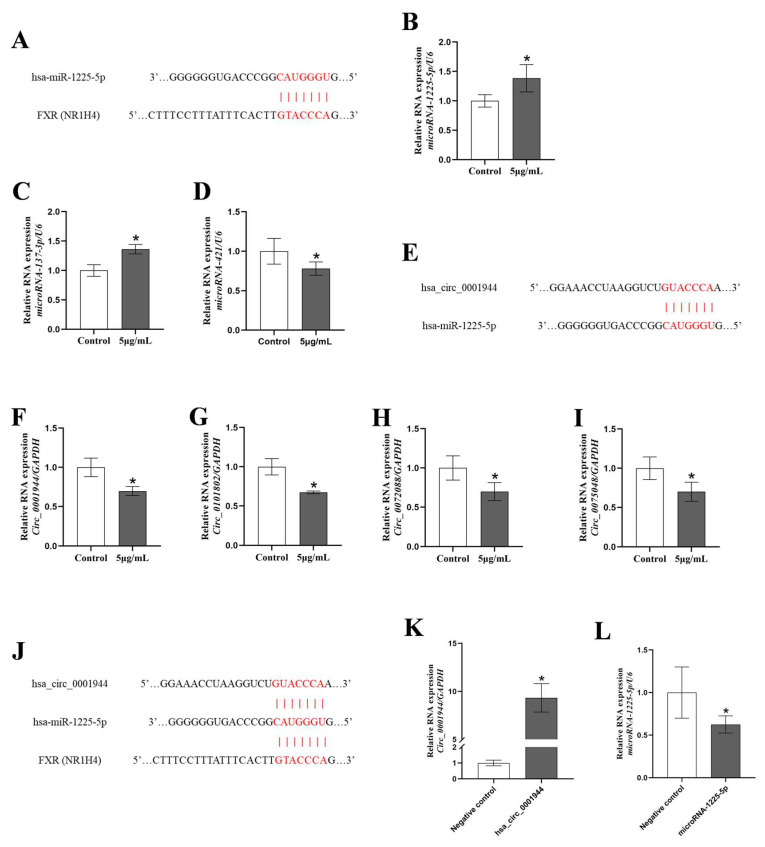
Screening of circRNA and construction of its overexpression cell. (**A**,**E**,**J**) The predicted binding sites and ceRNA network between FXR and miR-1225-5p and hsa_circ_0001944. (**B**–**D**) Expression levels of microRNA. (**F**–**I**) Expression levels of circRNA. (**K**,**L**) Expression levels of hsa_circ_0001944 and miR-1225-5p. * *p* < 0.05, compared to the control group.

To further investigate the mechanism of NiONPs-induced collagen deposition in LX-2 cells, we predicted the ceRNA network targeting FXR and the binding sites by bioinformatics (Figure 9A,E,J and Figure S4A,C,E,G). RT-qPCR results showed that 5 μg/mL NiONPs increased the levels of microRNA-1225-5p and microRNA-137-3p, and decreased the levels of microRNA-421 (Figure 9B–D). We selected microRNA-1225-5p, which has been reported to be related to the occurrence of liver injury [37,38] for further studies. We predicted circRNAs targeting microRNA-1225-5p (Figure 9E and Figure S4D,F,H); the expressions of hsa_circ_0101802, hsa_circ_0001944, hsa_circ_0072088 and hsa_circ_0072088 were decreased in LX-2 cells treated with 5 μg/mL NiONPs (Figure 9F–I). Then, we overexpressed hsa_circ_0001944, which had the same binding site as FXR and microRNA-1225-5p. Compared with the negative control group, the expression of hsa_circ_0001944 increased, and the expression of microRNA-1225-5p decreased (Figure 9K,L), indicating that the hsa_circ_0001944 overexpression plasmid was successfully constructed.

**Figure 10:**
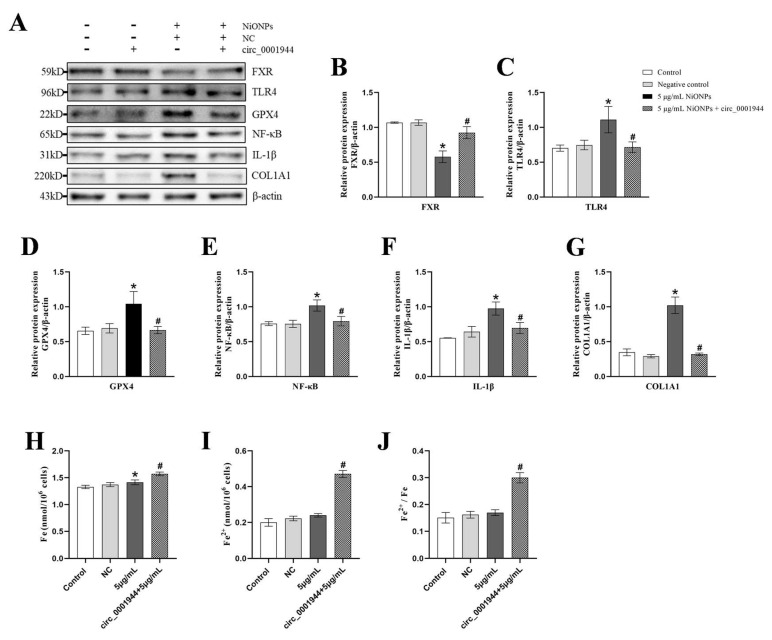
Overexpression of hsa_circ_0001944 alleviated the nickel oxide nanoparticles (NiONPs)-induced collagen in LX-2 cells through regulated Farnesol X receptor (FXR)/Toll-like receptor 4 (TLR4) signaling pathway and ferroptosis. (**A**–**G**) Protein expression levels of FXR (NR1H4), TLR4, GPX4, NF-κB, IL-1β and COL1A1. (**H**–**J**) Intracellular iron ion content detection. * *p* < 0.05, compared to the control group; ^#^ *p* < 0.05, compared with the 5 μg/mL NiONPs group.

Compared with the 5 μg/mL NiONPs group, the protein content of TLR4, GPX4, NF-κB, IL-1β and COL1A1 decreased, the level of FXR protein and the intracellular iron ion content and the proportion of ferrous ion increased treated with overexpression of hsa_circ_0001944 (Figure 10A–J). Those results indicated that overexpression of hsa_circ_0001944 alleviated the NiONPs-induced collagen deposition through the regulated FXR/TLR4 signaling pathway, increased the ferroptosis features and attenuated inflammation.

## 4. Discussion

This study proposed that hsa_circ_0001944 regulates the FXR/TLR4 pathway and ferroptosis, which might be a new mechanism for alleviating the activation of HSCs and collagen deposition caused by NiONPs. Activation of ferroptosis with Erastin inhibited NiONPs-induced collagen deposition in LX-2 cells. Inhibition of TLR4 with TAK-242 or activation of FXR with GW4064 alleviated the NiONPs-induced collagen in LX-2 cells by increasing the ferroptosis features and inhibiting inflammation. Overexpression of hsa_circ_0001944 alleviated the NiONPs-induced collagen in LX-2 cells by activating FXR, increasing the ferroptosis features, inhibiting TLR4 and the inflammatory response. These results provide a new idea for the cellular and molecular mechanisms of NiONPs-induced collagen deposition in HSCs.

The superior electrical, chemical, and magnetic properties of NiONPs particles have an increasing market demand in semiconductors, textiles, and other fields [2]. The widespread use of NiONPs increased the levels of nickel in the environment, while also increasing the exposure of populations [39]. Epidemiological studies showed a negative correlation between blood nickel level and liver function biomarkers in people exposed to the heavy metal nickel [40]. Toxicological studies showed that heavy metal nickel and their nanoparticles enter the organism and lead to liver damage [41]. Our previous study found that NiONPs induced liver fibrosis through activation of transforming growth factor beta 1 (TGF-β1)/Smad, JNK/c-Jun signaling pathways [6,9]. Activation and collagen deposition of HSCs promoted the development of liver fibrosis [42]. In this study, 5 μg/mL NiONPs induced collagen deposition, promoted the proliferation and migration ability in LX-2 cells. The development of collagen deposition of HSCs may be related to non-coding RNAs, nuclear receptor signaling and ferroptosis [10,11,12]. In this study, we found that overexpression of hsa_circ_0001944 by activating the FXR/TLR4 signaling pathway to inhibit inflammation and increase the ferroptosis features alleviated NiONPs-induced collagen deposition in LX-2 cells.

Liver injury is often accompanied by ferroptosis of hepatocytes, but the opposite phenomenon was shown in HSCs [13]. The GSH, GPX4 and SLC7A11 expression were decreased and ferroptosis was activated in CCl4-induced liver fibrosis mice [43], but the GPX4 and SLC7A11 expression were increased and ferroptosis signature was attenuated in LX-2 cells treatment with LPS [44]. Similar results were observed in our study, that the NCOA4 content increased and GPX4 content decreased in rats, while NCOA4 decreased and GPX4 increased in LX-2 cells. Targeting HSCs ferroptosis has demonstrated promising potential to alleviate collagen deposition [45]. We found that Erastin activated ferroptosis, reduced their proliferation and migration ability and decreased the expression of MMP2 and COL1A1 in NiONPs-treated LX-2 cells, which is consistent with previous studies reported to alleviate collagen deposition by activating ferroptosis of HSCs [16]. These findings indicated that activation of ferroptosis alleviates NiONPs-induced excessive collagen deposition.

The Toll-like receptor family, especially TLR4, plays a key role in the regulation of hepatocyte inflammation and collagen deposition [46]. Upregulation of TLR4 caused collagen deposition in hepatocytes by activating inflammation and increasing the levels of MMP2, MMP12 and COL1A1 [47,48]. We observed increased expression of TLR4 and inflammation-related markers (NF-κB, p-NF-κB, IL-1β, TNF-α) in both the liver tissue of rats and LX-2 cells treated with NiONPs, indicating that NiONPs caused collagen deposition by increasing TLR4 levels and activating inflammation. Inhibition of TLR4 in activated HSCs alleviated inflammation and reduced collagen deposition [49]. We observed that TAK-242, an inhibitor of TLR4, decreased the expression of NF-κB, IL-1β, MMP2 and COL1A1, and also inhibited the proliferation and migration ability of LX-2 cells. In addition, TLR4 has also been reported to participate in liver injury by upregulating hepcidin levels and increasing ROS and Fe^2+^ content to activating ferroptosis of hepatocytes [22]. Our results showed that TLR4 participates in the process of collagen deposition in hepatic stellate cells by regulating ferroptosis. TAK-242 decreased GSH and GPX4 levels and increased ROS and Fe^2+^ levels, and increased the ferroptosis features in LX-2 cells treated with NiONPs. Taken together, we concluded that downregulation of TLR4 alleviated NiONPs-induced LX-2 cell activation and collagen deposition by regulating ferroptosis and inhibiting inflammation.

The nuclear receptor FXR plays a crucial role in maintaining liver homeostasis [10]. Collagen formation was promoted by decreased FXR expression levels, and the activation of FXR inhibits the collagen deposition by reducing the activation of HSCs [50,51]. In this study, NiONPs decreased the level of FXR and increased the levels of MMP2 and COL1A1 in rat liver tissue and LX-2 cells, while FXR agonist GW4064 reduced cell viability and reversed the indicators of MMP2 and COL1A1 in LX-2 cells. These results indicated that NiONPs induced collagen deposition by downregulating FXR level in LX-2 cells. In addition, FXR suppressed inflammation response by inhibiting TLR4 [27]. We found GW4064 decreased the levels of TLR4, NF-κB and IL-1β in LX-2 cells treated with NiONPs, suggesting that activation of FXR alleviated collagen deposition by inhibiting TLR4 and inflammation. In addition, FXR alleviated liver injury by regulating ferroptosis of hepatocytes [28,52], and we observed the regulation of ferroptosis by FXR in hepatic stellate cells. After GW4064 treatment, we observed the decreasing GSH and GPX4 levels, increasing ROS and Fe^2+^ levels. Our results suggested that activation of FXR alleviated NiONPs-induced LX-2 collagen deposition by inhibiting TLR4 and the inflammatory response and increasing the ferroptosis features.

CircRNAs are noncoding RNAs with high abundance in mammalian cells, and exogenous compounds induced collagen deposition in hepatocytes by altering circRNAs levels [53]. TGF-β1 promoted excessive deposition of collagen in LX-2 cells by upregulating the circ_0044226 level [54]. We found that NiONPs decreased the expression of hsa_circ_0001944, while overexpression of hsa_circ_0001944 reduced the COL1A1 level in NiONPs-treated LX-2 cells, indicating that NiONPs induced collagen deposition by downregulating hsa_circ_0001944. CircRNAs regulated collagen deposition in HSCs by acting as microRNA sponges to increase the level of target genes [55,56]. TGF-β1 promoted collagen deposition by upregulating the level of hsa_circ_0009096 to adsorb miR-370-3p to increase transforming growth factor beta receptor 2 (TGFBR2) expression [57]. MicroRNA-1225-5p inhibited the viability of liver cancer cells by downregulating the level of NF-κB [37], but the relevant mechanism has not been reported in HSCs. We noticed that hsa_circ_0001944 and FXR shared the same binding sites with microRNA-1225-5p and overexpression of hsa_circ_0001944 decreased the expression of microRNA-1225-5p and increased the expression of FXR, indicating that hsa_circ_0001944 might regulate FXR levels by acting as a microRNA-1225-5p sponge in LX-2 cells. Meanwhile, we found that overexpression of hsa_circ_0001944 decreased the expression levels of TLR4, GPX4, NF-κB and IL-1β and increased the level of Fe^2+^ in LX-2 cells treated with NiONPs. We concluded that hsa_circ_0001944 alleviated NiONPs-induced collagen deposition by acting as a microRNA-1225-5p sponge to increase FXR expression levels, inhibit TLR4 and increase the ferroptosis features.

## 5. Conclusions

Our study provided evidence from cellular that non-coding RNAs, FXR/TLR4 signaling pathway, inflammatory response and ferroptosis were involved in NiONPs-induced collagen deposition in LX-2 cells. Our results indicated that hsa_circ_0001944 alleviated NiONPs-induced collagen deposition by activating FXR, inhibiting TLR4 and the inflammatory response, and increasing the ferroptosis features in LX-2 cells. In addition, our findings suggest that hsa_circ_0001944, FXR, TLR4 and ferroptosis can be used as possible intervention targets for collagen deposition in hepatocytes in the future. These findings provide a research foundation for future studies exploring the new methods of prevention and treatment in liver fibrosis.

## Figures and Tables

**Table 1:** Primer sequences of qPCR.

**Gene Names**	**Category**	Primer Sequence (5′-3′)
*hsa-miR-1225-5p*	miRNA	F-AATGTCGTGGGTACGGCCCA R-ATCCAGTGCAGGGTCCGAGG RT-GTCGTATCCAGTGCAGGGTCCGAG GTATTCGCACTGGATACGACCCCCCCAC
*hsa-miR-421*	miRNA	F-CGCGGCCATCAACAGACATTAAT R-ATCCAGTGCAGGGTCCGAGG RT-GTCGTATCCAGTGCAGGGTCCGAG GTATTCGCACTGGATACGACGCGCCC
*hsa-miR-137-3p*	miRNA	F-CGCGCGTTATTGCTTAAGAATAC R-ATCCAGTGCAGGGTCCGAGG RT-GTCGTATCCAGTGCAGGGTCCGAG GTATTCGCACTGGATACGACCTACGC
*hsa_circ_0001944*	circRNA	F-GAGAGGAGATACTTTATGAGGAGACTAAGG R-GCAAGCCAGGTACAGTCTTGTG
*hsa_circ_0101802*	circRNA	F-GAAGAATGTGTCCAGCTACCCA R-CTGCTTTCTCTCTTCTTCTGCC
*hsa_circ_0072088*	circRNA	F-ATGGTCTGCAGTCCTGTGTG R-TGGATAAATGGTGGCATGTTT
*hsa_circ_0075048*	circRNA	F-ATGAAGATCCCGCTGAACAA R-CAGACTGACGTCGATCTTGC
*GAPDH*	mRNA	F-TATGACAACAGCCTCAAGAT R-AGTCCTTCCACGATACCA
*U6*	snRNA	F-CTCGCTTCGGCAGCACA R-AACGCTTCACGAATTTGCGT

**Table 2:** The antibody information.

Name	Catalog Number	Dilution Ratio	Source
NR1H4 (FXR)	#31507	1:1000	Signalway Antibody
NR0B2 (SHP)	#32460	1:1000	Signalway Antibody
TLR4	#35463	1:1000	Signalway Antibody
MyD88	#32107	1:1000	Signalway Antibody
NCOA4	#32981	1:1000	Signalway Antibody
GPX4	#32506	1:1000	Signalway Antibody
NF-κB p65	#6956	1:1000	Cell Signaling Technology
p-NF-κB p65	#3033	1:1000	Cell Signaling Technology
IL-1β	#12242	1:1000	Cell Signaling Technology
TNF-α	#3707	1:1000	Cell Signaling Technology
MMP2	#29090	1:1000	Signalway Antibody
COL1A1	#81375	1:1000	Cell Signaling Technology
β-actin	#21338	1:5000	Signalway Antibody
Goat Anti-Rabbit IgG Secondary Antibody HRP Conjugated	#L3012	1:10,000	Signalway Antibody
Goat Anti-Mouse IgG Secondary Antibody HRP Conjugated	#L3032	1:10,000	Signalway Antibody

## Data Availability

Data will be made available on request.

## Supplementary Materials

The following supporting information can be downloaded at https://www.mdpi.com/article/10.3390/toxics13040265/s1, Figure S1: Construction of plasmid overexpressing hsa_circ_0001944; Figure S2: GO functional annotation analysis; Figure S3: (A–C) Scratch healing test (×100, n = 3); Figure S4: Bioinformatics prediction; Table S1: The hsa_circ_0001944 primer sequence.
