# Supplementary material for: Hsa_circ_0001944 Regulates FXR/TLR4 Pathway and Ferroptosis to Alleviate Nickel Oxide Nanoparticles-Induced Collagen Formation in LX-2 Cells
Source: Toxics. 2025 Mar 31;13(4):265. doi: 10.3390/toxics13040265 (PMC12031114; doi:10.3390/toxics13040265)
Supplement: Supplementary file 1 [file toxics-13-00265-s001.zip › toxics-3496226-supplementary.pdf]

## *Supporting information*

**Table S1** The hsa_circ_0001944 primer sequence.

| Gene ID | Seq (5’ → 3’) |
|---|---|
| has_circ_0001944-F | ACTCCCCACCATCACTTTTTAGAGACTAAGGTGTCAGTATGTTC |
| has_circ_0001944-R | AACTTGGGAAATTCTTTTGTACCCTCATAAAGTATCTCCTCTCTTTAAG |

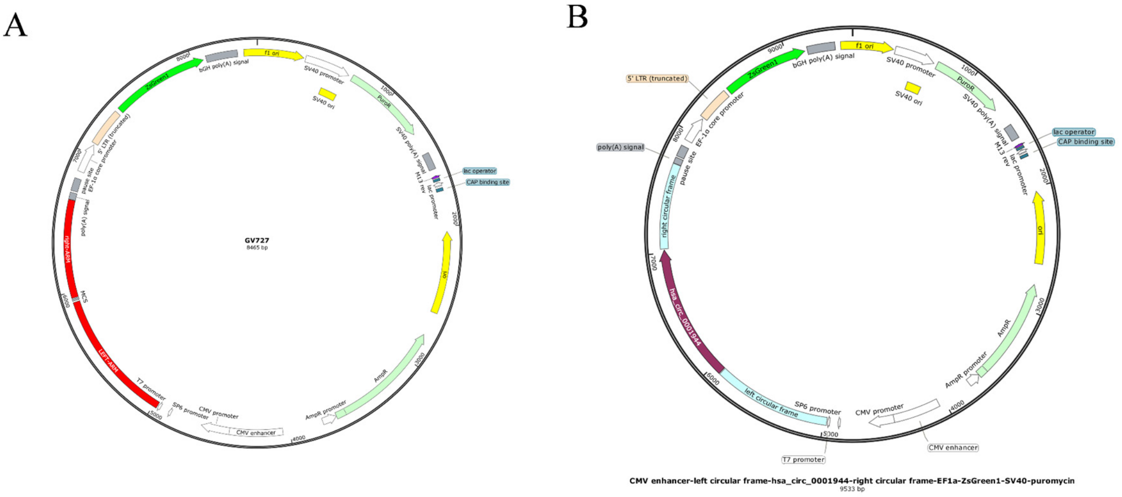


**Figure S1.** Construction of plasmid overexpressing hsa_circ_0001944. (A) Schematic of the overexpression vector GV727. (B) Schematic of the hsa_circ_0001944 overexpression plasmid.

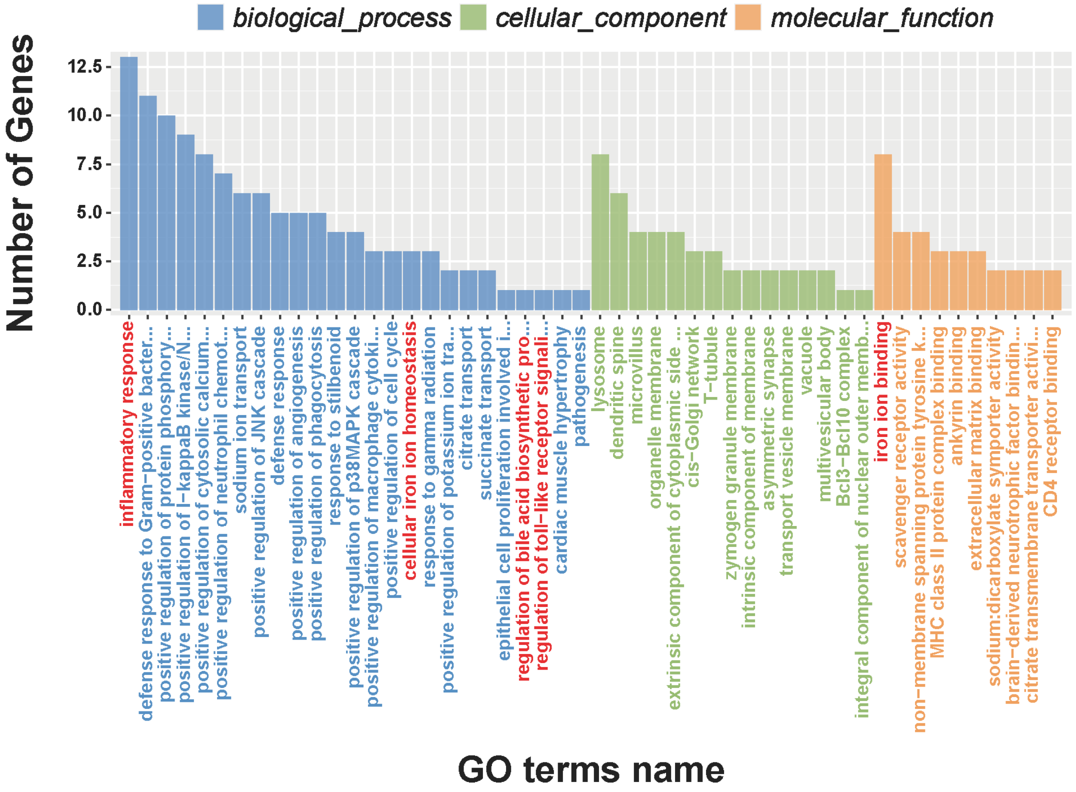


**Figure S2.** GO functional annotation analysis.

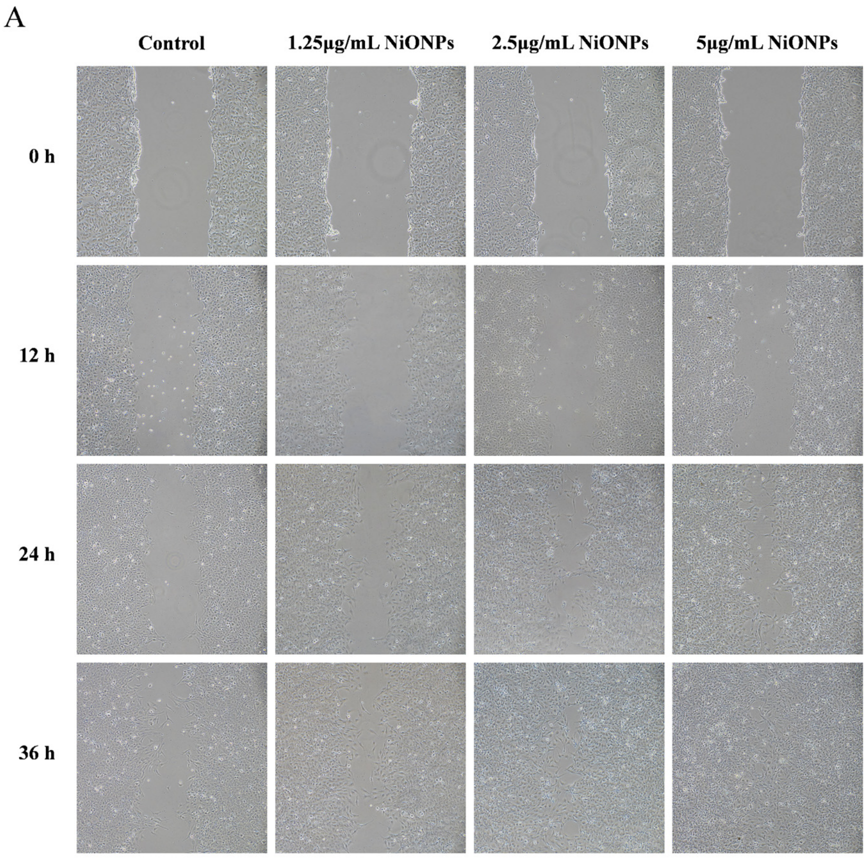
A
Control
1.25μg/mL NiONPs
2.5μg/mL NiONPs
5μg/mL NiONPs
0 h
12 h
24 h
36 h

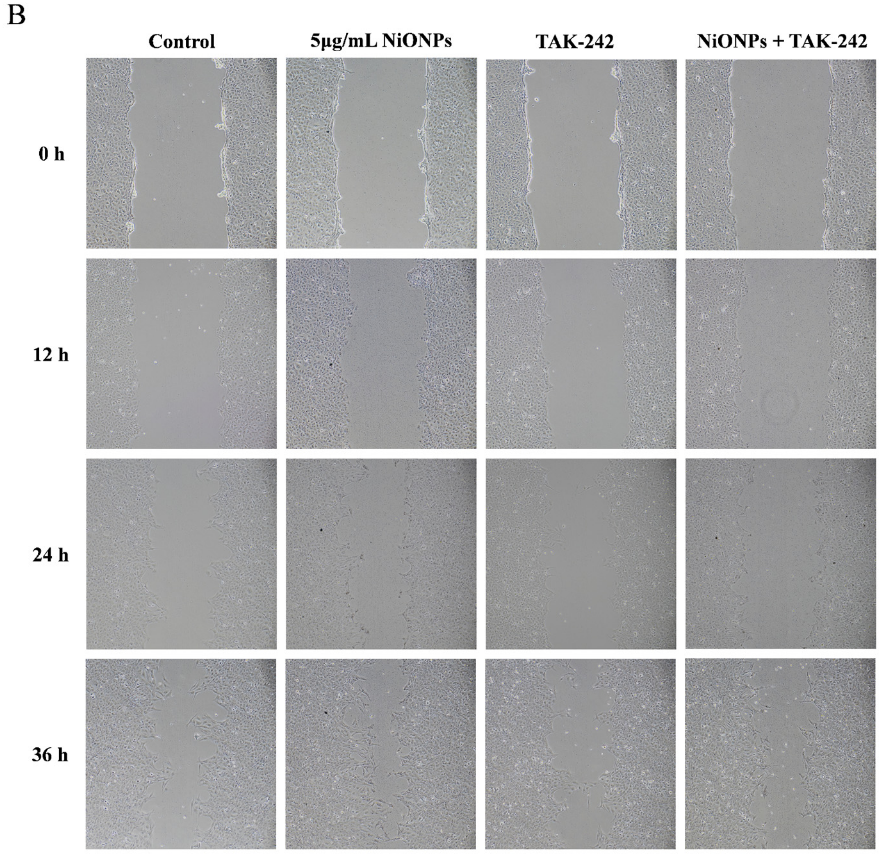
B
Control
5μg/mL NiONPs
TAK-242
NiONPs + TAK-242
0 h
12 h
24 h
36 h

C

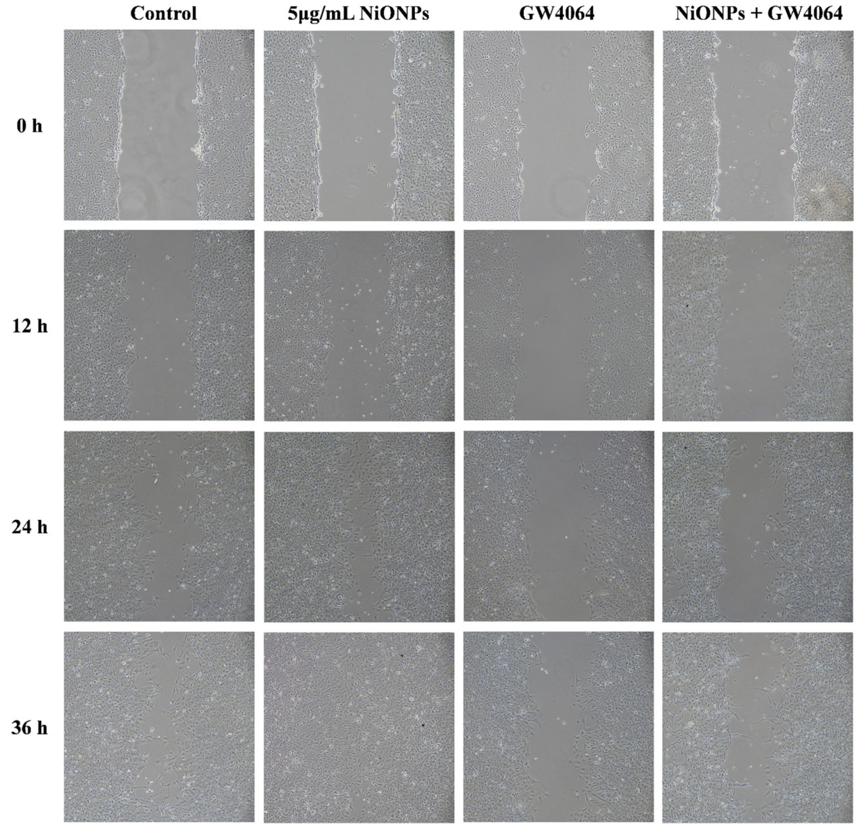


**Figure S3.** (A-C) Scratch healing test (×100, n=3).

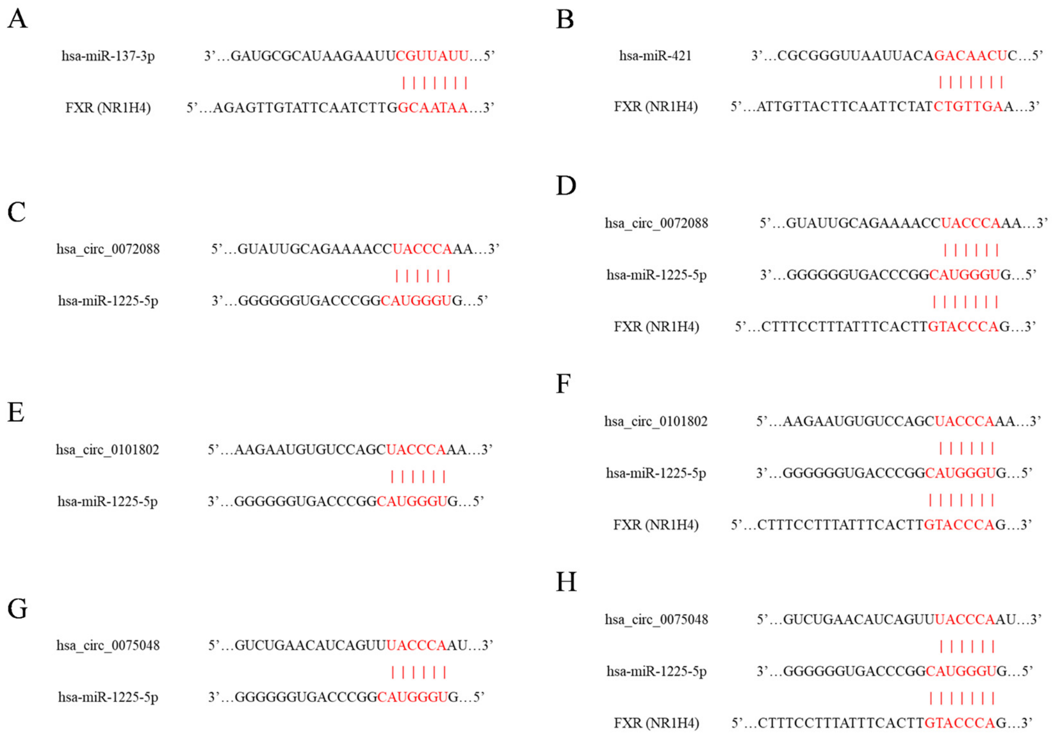


**Figure S4.** Bioinformatics prediction. (A, B, C, E, G) The predicted binding sites. (D, F, H) The predicted ceRNA networks.
